# Supplementary material for: An equivariant Bayesian convolutional network predicts recombination hotspots and accurately resolves binding motifs
Source: Bioinformatics. 2018 Nov 27;35(13):2177–84. doi: 10.1093/bioinformatics/bty964 (PMC6596897; doi:10.1093/bioinformatics/bty964)
Supplement: bty964_Supplementary_Data.pdf [file bty964_supplementary_data.pdf]

Supplementary Materials

November 13, 2018

A Network Topologies

A.1 Baseline Asymmetric Networks

We performed a hyperparameter search for the best asymmetric topologies for each of the two datasets, not allowing for equivariance or MC dropout layers.

Simulation topology

Layers

- Input 1000 $\times 4$
- Convolutional Layer (12 filters, length 14)
- Elu activation
- Global Spatial Max Pooling
- 2 Output neurons
- Softmax

Regularization

- L2 norm 0

Recombination topology

Layers

- Input 997 $\times 4$
- Convolutional Layer (16 filters, length 30)
- Elu activation
- Spatial Max Pooling (Size 8, Stride 8)
- Convolutional Layer (16 filters, length 4)
- Elu activation
- Global Spatial Max Pooling
- 2 Output neurons
- Softmax

Regularization

- L2 norm 0.0003

Networks obtained for the data augmentation optimization were identical, except that the optimal L2 regularization parameter was 0 for the recombination dataset, and the number of filters was doubled to 32. Note that when we followed the procedure in A.2, we used an equivariant Bayesian network with this 32 filters when comparing against augmented data.

A.2 Bayesian Equivariant Networks

For each data set, we performed independent hyperparameter searches for the case of (1) equivariant networks, (2) Bayesian networks, and (3) equivariant Bayesian networks, as described in the main text. Note that for the case of equivariant networks we kept the same number of filters but tied their parameters to achieve equivariance, effectively halving the number of filters and parameters.

We summarise the results by giving the explicit networks for case (3); the optimal networks for cases (1) and (2) turned out to be the same as those for

case (3) except for dropping the relevant layers/features (underlined), and changing "Equivariant MC dropout" into "MC dropout" where relevant.

Simulation topology

Layers

- Input 1000 $\times 4$
- Equivariant Convolutional Layer (12 filters, length 14)
- Elu activation
- Reverse Complement Mean Pool
- Equivariant MC dropout (p = 0.1)
- Global Spatial Max Pooling
- 2 Output neurons
- Softmax

Regularization

- L2 norm 0

Recombination topology

Layers

- Input 997 $\times 4$
- Equivariant Convolutional Layer (16 filters, length 30)
- Elu activation
- Equivariant MC Dropout (p=0.1)
- Spatial Max Pooling (Size 8, Stride 8)
- Equivariant Convolutional Layer (16 filters, length 4)
- Elu activation
- Equivariant MC Dropout (p=0.1)
- Global Spatial Max Pooling
- Reverse Complement Sum Pooling
- 2 Output neurons
- Softmax

Regularization

- L2 norm 0

Note that for both datasets we initialized the output layer as per section 3.8 in the main text.

A.3 Medians AUROCS for data presented in Fig. 3

Dataset	Baseline	MC dropout	Equivariant	Equivariant MC dropout
Recombination	0.684	0.693	0.690	0.706
Simulated	0.616	0.620	0.627	0.634

A.4 Accuracies from Fig. 4

DEMO CNN	13mer	DEMO CNN RNN	DEMO RNN	Equivariant Bayesian	H3H4me3
0.533	0.540	0.598	0.603	0.663	0.665

B ReLU activation function performs worse than ELU

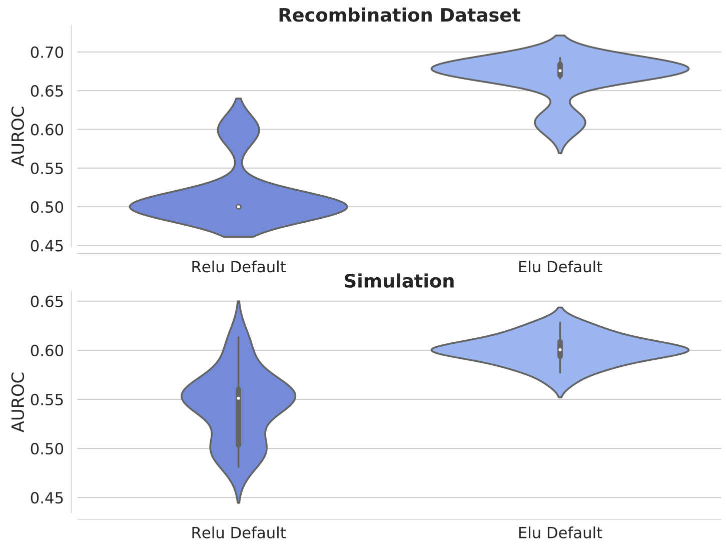

Figure 1: A comparison of convergence accuracy for 25 runs of the best asymmetric architecture with standard initialization for ReLU and ELU activation functions for both datasets

One phenomenon that we observe for our dataset is that the choice of activation function is important in determining convergence behaviour. We noticed that the standard ReLU function resulted in poor convergence on both datasets. Figure 1 shows the convergenced accuracy plotted for the

best asymmetric topologies presented in A, with a default weight initialization and a choice of ReLU and ELU activation functions. We see from this figure considerable outperformance for the ELU activation function (with qualitatively similar results for the shifted ReLU or SReLU). The behaviour observed here is qualitatively similar to what we saw for the DeMo CNN model, and an implementation of a DeepBind like network, and suggest that this choice of activation function goes some way to explaining the variety of convergence accuracy seen here.

C Custom Initialization helps ReLU to converge reliably in both datasets

As an alternative approach to using a different activation function to ReLU, we also investigated the effects of a custom initialization of the output layer on the convergence accuracy. For our topologies we had 2 output neurons and a Softmax activation, so the final layer requires a weight matrix of $[N, 2]$, and 2 bias neurons. We initialized this weight matrix to ones, and the biases to $[1, -1]$. Despite no theoretical grounding of this initialization we found that it solved the convergence issues across a number of topologies in both, and other simulated, datasets.

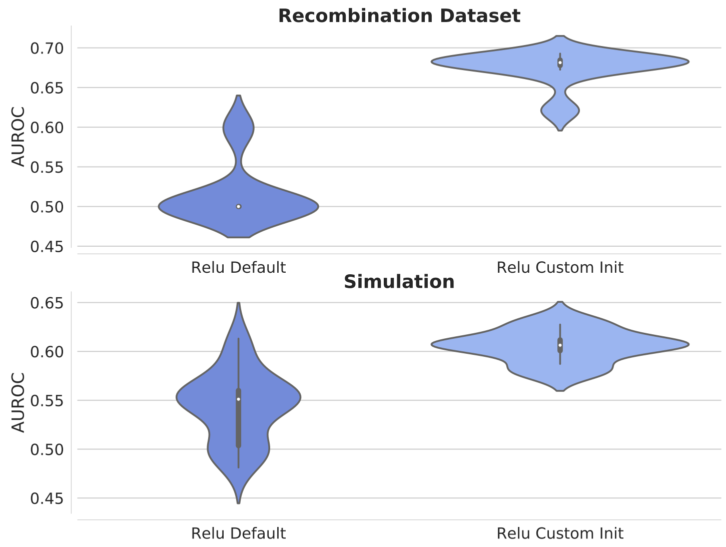

Figure 2: A comparison across both datasets for the best asymmetric architecture with ReLU activation with and without unit output initialization

D ReLU activation does not consistently converge from local optimum initialization

As a further test of the robustness of the relu activation function, we tried a partial initialisation of the filters on the simulated data where we know that there are 2 driving motifs and their reverse complements, ATF4 and EGR1. In order aid the network in convergence, two of the filters equal to the PWM of ATF4 and it's reverse complement, and then trained an Equivariant network from this point to observe the classification accuracy at convergence. Each column of the PWM sums to 1. The results of this analysis are in figure 3. Strikingly, we found the ReLU activation function struggled to converge from this seemingly favourable initialization, and was almost always unable to find the EGR1 motif. By way comparison we built an identical network, swapping only the activation functions for either an Exponential Linear Unit (ELU) of a Shifted ReLU (SReLU). Both of these functions performed substantially better, and were much more readily able to detect the remaining signal.

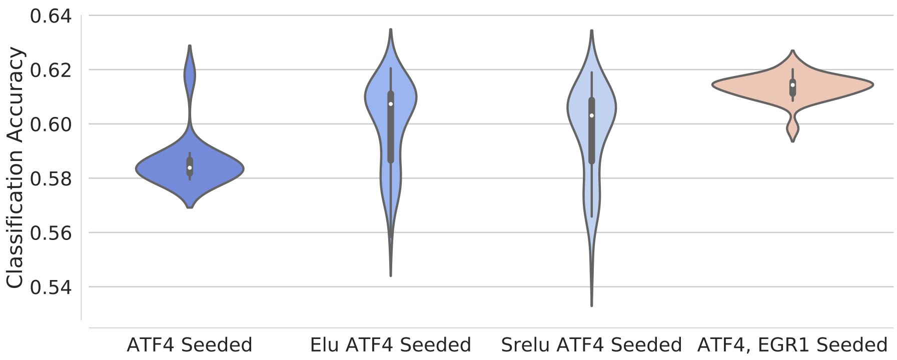

Figure 3: Convergence accuracy for Equivariant network with ReLU, ELU and SReLU activation functions on the simulated dataset, built from 50 trial runs. We seeded the filter bank with PWMs for the ATF4 motif, and its reverse complement and then trained the network to convergence

E Bayesian Equivariant Network outperforms data augmentation

An alternative and heavily used approach to improve the quality of inference in deep learning problems is one of data augmentation: perturbing the input data in such a way that does not semantically alter the subject of the image. In image recognition, this might be adding low magnitude white noise, or rotating or translating the image in such a way as keep the subject of the image in full view. This approach reduces over-fitting by creating more training data, and can be used to generate many orders of magnitude more examples for each problem. In genomics, a key perturbation that can be applied to the sequences is reverse complementing them, thus doubling the number of training examples. We compare the results of the Bayesian equivariant neural network to the an asymmetric network optimized independently for augmented data as per section 2.10 of the main text, with Bayesian dropout allowed. Here, the number of filters in both networks are identical, and the only difference in terms of layers is the reverse complement pooling after the convolutional layers. Here, we observe statistically significantly better performance for the simulated dataset, and indistinguishable performance for the recombination dataset. Results presented in figure 4.

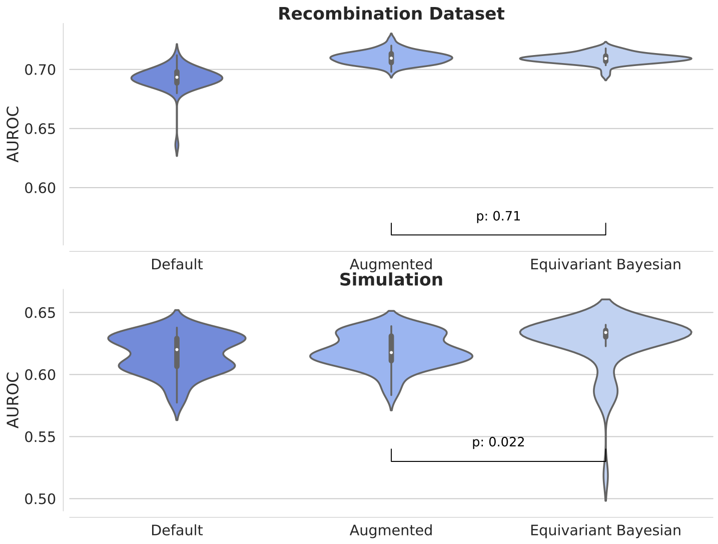

Figure 4: Comparison between the best discovered asymmetric network optimized for augmented data and the best equivariant networks trained on the unaugmented data

F Batch Norm in this regime

We compare the convergence of the asymmetric networks in section A.1 when they implement appropriate batch normalization layers. Results are presented in 5. Batch norm does appear to improve the networks for ReLU activation, but appears marginally deleterious for the ELU networks. We compare these networks to the ELU network with the custom initialization described in the main text (far right of 5) and find that they perform qualitatively worse in both datasets.

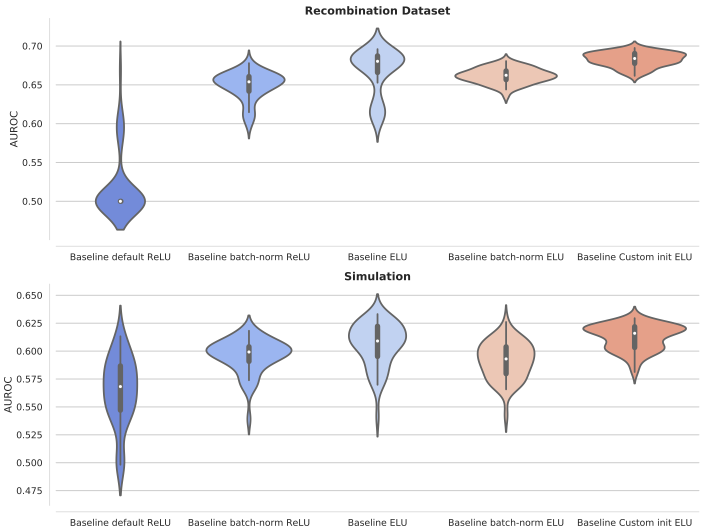

Figure 5: Examination of the effect of batch normalization on the chosen networks with default initialization with both ReLU and ELU activation functions.

G Model comparison in the low-data regime

We performed a robustness experiment to determine how the specified networks performed when the data is reduced. We compared the networks with weight equivariance but no MC dropout (the 3rd network accross in figure 3 in the main text, which is the same as the networks in A.2 without dropout) to the networks in A.1, in the regime where we remove first 50% and then 75% of the data. For the recombination network, we find that the equivariant networks here provide increased convergence accuracy in comparison to the asymmetric versions, suggesting that this approach may be even more useful when there are fewer training examples than the c. 26,000 we have in our datasets. (Note that this isn't the same number of sequences given in the main text as we split into training, validation and testing tranches.

For the simulated data, we observed a bimodal distribution with dataset size reduction, as the network struggles to converge reliably, possibly due to the regularizing effect of both dropout and weight equivariance in the

filters. What is striking, is that the best results still belong to the equivraint Bayesian network, but this is dragged down by the increased numbers of failed convergences. Note that these results represent an incredibly noise domain with a convergence auroc of only around 0.54. Results in 7.

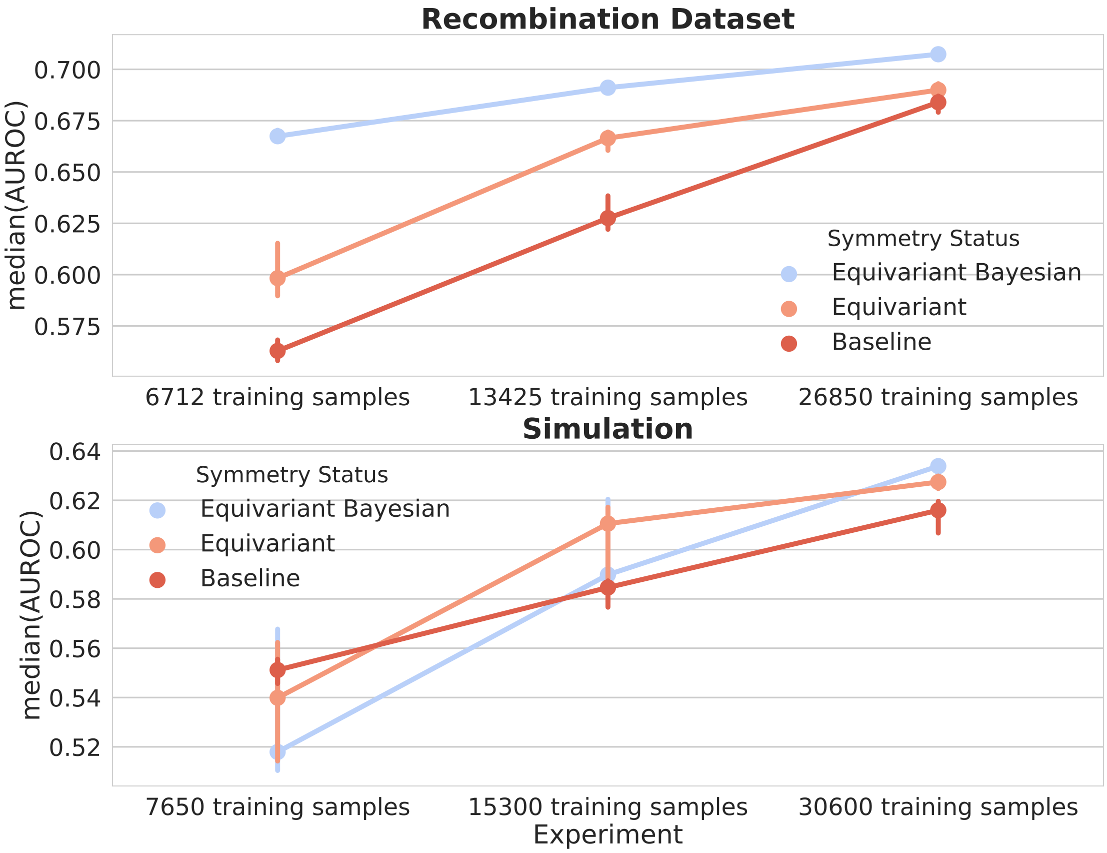

Figure 6: Comparison of the mean test AUROC for equivariant vs asymmetric networks show increased accuracy as train set size is reduced.

H Homer Motif Results

We present the results of running HOMER (Heinz 2010) to discover differentially expressed motifs between hotspots an coldspots. We ran the software using default parameters, except for the target motif length, which we set to 22 in order to attempt to recover the sparse motif we found using the neural network approach. Whilst it is possible that another parameter setting would yield better results, it is difficult to tune these parameters in an analogous fashion to performing a hyperparameter search due to the lack of a cost function to optimize. long time for each run to execute.

Whilst we fail to generate the motif of interest we do generate 1 interesting result: Specifically, motifs 1-3 contain within in them stretches that correspond to the canonical PRDM9 13mer binding sites that was discovered in Myers (2008). This is an unsurprising find, as it was discovered on a similar dataset curated from recombination hotspots using an exhaustive search. Aside from this, we only find motifs 5,9 and 12 with substantial presence, however they bear little resemblance to the discovered sparse motif we identify.

Rank	Motif	P-value	log P-pvalue	% of Targets	% of Background
1	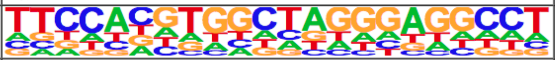	1e-200	-4.607e+02	0.80%	0.01%
2	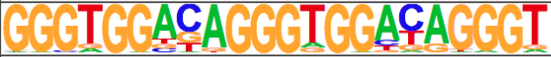	1e-145	-3.356e+02	62.76%	53.13%
3	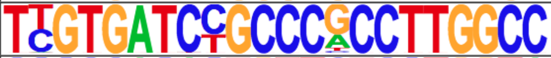	1e-116	-2.678e+02	1.86%	0.38%
4	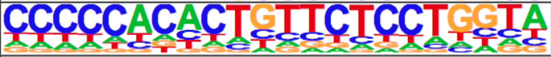	1e-64	-1.491e+02	0.34%	0.01%
5	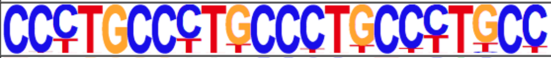	1e-53	-1.238e+02	36.39%	30.91%
6	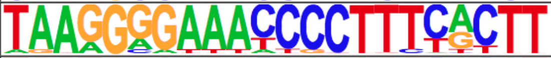	1e-51	-1.191e+02	0.29%	0.01%
7	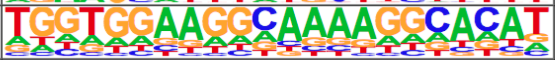	1e-51	-1.188e+02	0.24%	0.01%
8	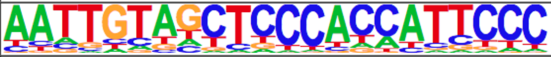	1e-40	-9.411e+01	0.36%	0.03%
9	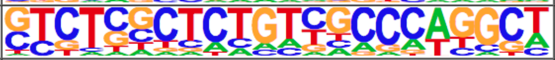	1e-32	-7.444e+01	65.12%	60.76%
10	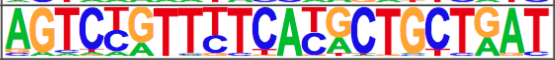	1e-31	-7.185e+01	1.79%	0.85%
11	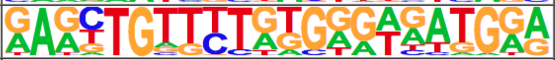	1e-14	-3.445e+01	0.10%	0.01%
12	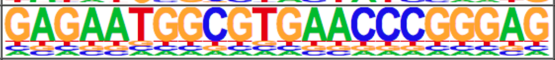	1e-12	-2.793e+01	75.44%	73.10%

Figure 7: Top motifs found using a discriminate search with HOMER. Running on a single 1.7 GHz Intel Core i7 core, the results presented were generated in c. 24 hrs

I Hyperparameter Search Space

I.1 Recombination

Parameter	Options
# Convolutional layers	1 . . . 5
Filter length of 1st Convolution layer	10, 15, 20, 30
Filter lengths of internal layers	4, 8, 12
Max Pool sizes (stride is always pool size)	4, 8, 12
Number of filters	8, 16, 32, 64
L2 regularization	0, 0.0001, 0.0003, 0.001
lrate	0.1, 0.01, 0.001, 0.0001

Networks sampled at random 1000 times and trained to convergence with ADAM

I.2 Simulation

Parameter	Options
# Convolutional layers	1
Filter length of 1st Convolution layer	14
Number of filters	4, 6, 12
L2 regularization	0, 0.0001, 0.0003, 0.001
lrate	0.1, 0.01, 0.001, 0.0001

Networks sampled at random 200 times and trained to convergence with ADAM
